# Antigiardial Activity of Podophyllotoxin-Type Lignans from *Bursera fagaroides* var. *fagaroides*

**DOI:** 10.3390/molecules22050799

**Published:** 2017-05-13

**Authors:** Filiberto Gutiérrez-Gutiérrez, Ana María Puebla-Pérez, Sirenia González-Pozos, José Manuel Hernández-Hernández, Armando Pérez-Rangel, Laura Patricia Alvarez, Gabriela Tapia-Pastrana, Araceli Castillo-Romero

**Affiliations:** 1 Departamento de Química y Farmacobiología, Universidad de Guadalajara, Guadalajara 44430, Jalisco, Mexico; fili_gutierrez@hotmail.com (F.G.-G.); ampueblap@yahoo.com.mx (A.M.P.); 2 Unidad de Microscopía Electrónica LaNSE, Centro de Investigación y Estudios Avanzados del Instituto Politécnico Nacional, Ciudad de México 07360, Mexico; sgonzale@cinvestav.mx; 3 Departamento de Biología Celular, Centro de Investigación y Estudios Avanzados del Instituto Politécnico Nacional, Ciudad de México 07360, Mexico; manolo@cell.cinvestav.mx (J.H.-H.); rangelarm@yahoo.com (A.C.-R.); 4 Centro de Investigaciones Químicas, Universidad Autónoma del Estado de Morelos, Morelos 62209, Mexico; lalvarez@uaem.mx; 5 Laboratorio de Investigación Biomédica, Hospital Regional de Alta Especialidad de Oaxaca, Oaxaca 71256, Mexico; gabrielatapiapastrana@gmail.com; 6 Departamento de Microbiología y Patología, Universidad de Guadalajara, Guadalajara 44340, Jalisco, Mexico

**Keywords:** *Bursera fagaroides*, lignans, antiprotozoal activity, *Giardia lamblia*

## Abstract

Giardiasis, a diarrheal disease, is highly prevalent in developing countries. Several drugs are available for the treatment of this parasitosis; unfortunately, all of them have variable efficacies and adverse effects. *Bursera fagaroides* has been known for its anti-inflammatory and antidiarrheal properties in Mexican traditional medicine. We investigated the in vitro anti-giardial activities of four podophyllotoxin-type lignans from *Bursera fagaroides* var. *fagaroides*, namely, 5′-desmethoxy-β-peltatin-A-methylether (5-DES), acetylpodophyllotoxin (APOD), burseranin (BUR), and podophyllotoxin (POD). All lignans affected the *Giardia* adhesion and electron microscopy images revealed morphological alterations in the caudal region, ventral disk, membrane, and flagella, to different extents. Only 5-DES, APOD, and POD caused growth inhibition. Using the Caco-2 human cell line as a model of the intestinal epithelium, we demonstrated that APOD displayed direct antigiardial killing activity and low toxicity on Caco-2 cells. This finding makes it an attractive potential starting point for new antigiardial drugs.

## 1. Introduction

*Giardia lamblia* is one of the most ancient eukaryotes known. It is a flagellated protozoan parasite that infects the small intestine of humans and other mammals producing the diarrheal disease giardiasis [1]. This infection has worldwide distribution and is highly prevalent in developing countries [2,3,4,5]; it affects young children, primarily. Several drugs are available for treatment; unfortunately, all of them present variable efficacies and undesirable side effects [6,7,8,9], and some strains of *Giardia* have shown resistance towards common drugs. For these reasons, the search for new therapies with fewer side effects and better effectiveness is of great significance. Several natural products have been tested searching for new antigiardial therapies [10,11,12]. Podophyllotoxin, an aryltetralin-type lignan isolated mainly from *Podophyllum peltatum*, exhibited important biological activities, such as anti-tumor effects. It inhibits the cell growth by microtubule disassembly of the mitotic spindle apparatus [13,14,15,16]. Recently, the podophyllotoxin antigiardial activity was demonstrated—it inhibits the growth and causes cell death [17]—but the podophyllotoxin molecular mechanisms of action and its effects on trophozoites morphology are still unknown. On the other hand, *Bursera fagaroides* (family Burseraceae), known in Mexico as “Iztac quauhxiotl”, “Palo Xixote”, and “Cuajiote amarillo”, is an aromatic tree of about 3–6 m tall distributed from the Southwestern United States of America to the Isthmus of Tehuantepec in Mexico [18]. It has been known for its anti-inflammatory, anti-cancer, and antidiarrheal properties in Mexican traditional medicine [19,20,21,22,23]. In addition, it was demonstrated that an ethanolic extract of this species affected the *Entamoeba histolytica* growth and inhibited the activity of the enzyme ornithine decarboxylase [24]. 

*B. fagaroides* extracts have been investigated in order to identify the secondary metabolites responsible for the biological activities present in this plant. Currently, around fourteen podophyllotoxin-type lignans from *B. fagaroides* have been isolated and characterized, including podophyllotoxin; some of them have shown significant cytotoxic activity in several cancer cell lines [22,25,26]. However, their effects on parasites have been poorly examined to date. In this study, we analyzed the effect of burseranin (BUR), 5′-desmethoxy-β-peltatin-A-methylether (5-DES), acetylpodophyllotoxin (APOD), and podophyllotoxin (POD) on *Giardia lamblia* trophozoites. Our results showed that all of the tested lignans affected the growth and adhesion of *Giardia* trophozoites to different extents. Concomitantly, microscopy images revealed significant morphological alterations after lignan treatment, except for BUR. In addition, we demonstrated that APOD displayed direct antigiardial killing activity and low toxicity on Caco-2 cells.

## 2. Results

### 2.1. Dose-Dependent Effect of Podophyllotoxin-Type Lignans from *Bursera fagaroides* on *Giardia lamblia* Trophozoite Growth and Viability

**Figure 1:**
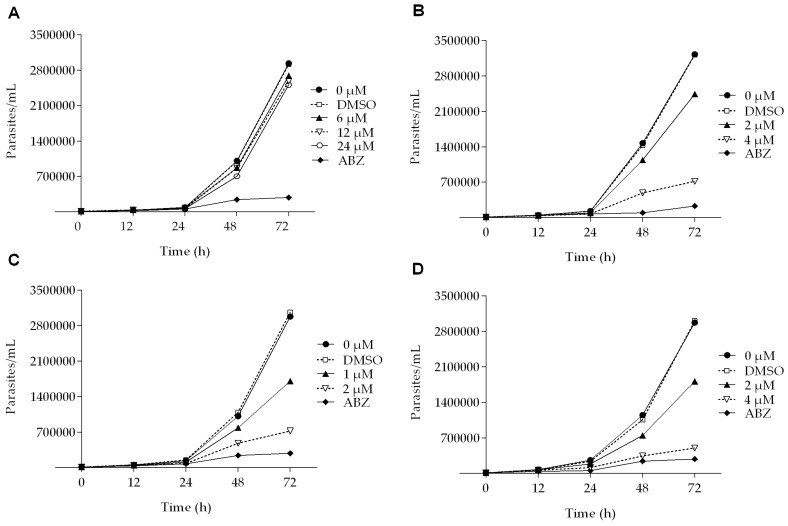
Growth kinetics of *Giardia lamblia* trophozoites in the presence of (**A**) burseranin, (**B**) 5′-desmethoxy-β-peltatin-A-methylether, (**C**) acetylpodophyllotoxin, and (**D**) podophyllotoxin.

**Figure 2:**
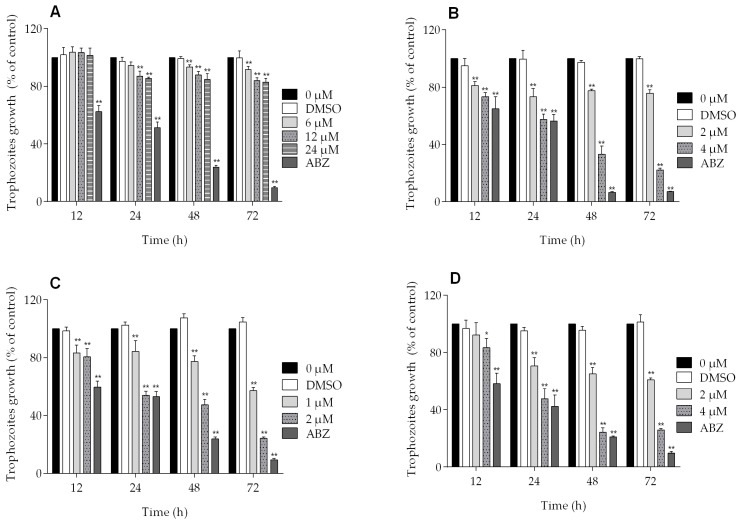
Percent of growth inhibition of *Giardia lamblia* trophozoites in the presence of (**A**) burseranin, (**B**) 5′-desmethoxy-β-peltatin-A-methylether, (**C**) acetylpodophyllotoxin, and (**D**) podophyllotoxin (*p < 0.005, **p < 0.0001).

**Figure 3:**
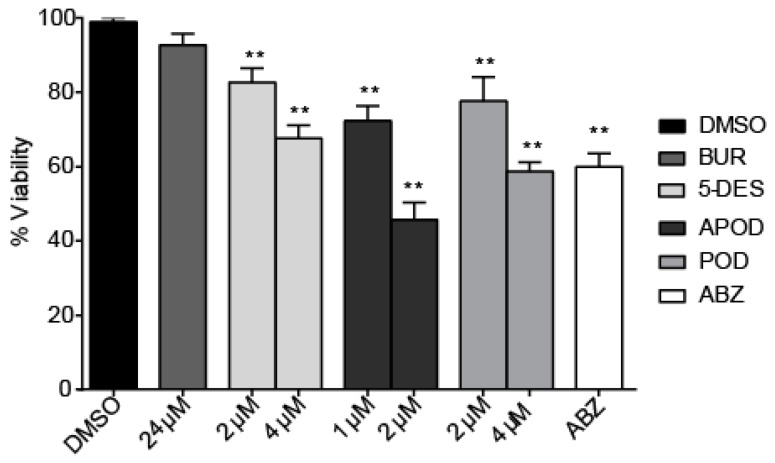
Effect of burseranin (BUR), 5′-desmethoxy-β-peltatin-A-methylether (5-DES), acetylpodophyllotoxin (APOD), podophyllotoxin (POD), and albendazole (ABZ) on *Giardia lamblia* trophozoite viability after 24 h of treatment: (**p < 0.0001).

All of the podophyllotoxin-type lignans tested here inhibited the growth of *G. lamblia* trophozoites to different extents. The inhibitory effects, whose kinetics are shown in Figure 1, revealed a dose-dependent inhibition, with an IC_50_ value of 4.53 μM for 5-DES, 2.12 μM for APOD, and 3.88 μM for POD (Table 1). BUR caused only a moderate inhibition effect on parasite growth (Figure 1A). The maximal inhibitory effects were observed after 72 h of incubation; at 24 μM, BUR decreased cell growth by 17% (Figure 2A), treatment with 4 μM of 5-DES or POD decreased cell growth by 77% and 75%, respectively (Figure 2B,D), whereas treatment with 2 μM APOD decreased cell growth by 75% (Figure 2C). In addition, the percentage of viable parasites was determined using a trypan blue dye exclusion assay. The incubation of trophozoites with 4 µM of 5-DES or POD caused a decrease in viability percentages of parasites of 67% and 58%, respectively. Treatment with 2 µM APOD resulted in cell viability of 45%, suggesting that APOD is more active against *Giardia* trophozoites. The group treated with BUR showed no significant changes (Figure 3). Dimethyl sulfoxide (DMSO)-treated cells did not exhibit any significant differences compared with untreated cells.

### 2.2. Podophyllotoxin-Type Lignans Affect the Adhesion of *Giardia lamblia* Trophozoites

**Figure 4:**
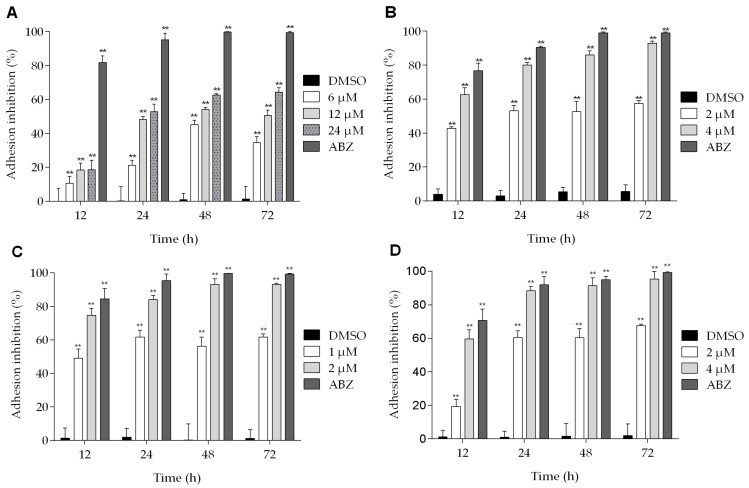
Effect of podophyllotoxin-type lignans on *Giardia lamblia* trophozoite adhesion after treatment with burseranin (**A**), 5′-desmethoxy-β-peltatin-A-methylether (**B**), acetylpodophyllotoxin (**C**), and podophyllotoxin (**D**) (**p < 0.0001).

The effects of podophyllotoxin-type lignans on the adherence of trophozoites are shown in Figure 4. All of the lignans tested have an inhibitory effect on *Giardia* adhesion to different extent, and the maximum effect was observed after 72 h of treatment. At this time, BUR reduced the adhesion of parasites by 64% (Figure 4A). On the other hand, the effect of 5-DES and POD at 4 μM was similar (inhibition of 92% and 95%, respectively) (Figure 4B,D). Meanwhile, APOD caused a more dramatic effect: with 1 μM, the adhesion was reduced by nearly 50% at 12 h, and, with 2 μM, at 72 h the maximal inhibition was observed (93%) (Figure 4C).

### 2.3. Podophyllotoxin-Type Lignans Affect the Morphology of *Giardia lamblia* Trophozoites 

**Figure 5:**
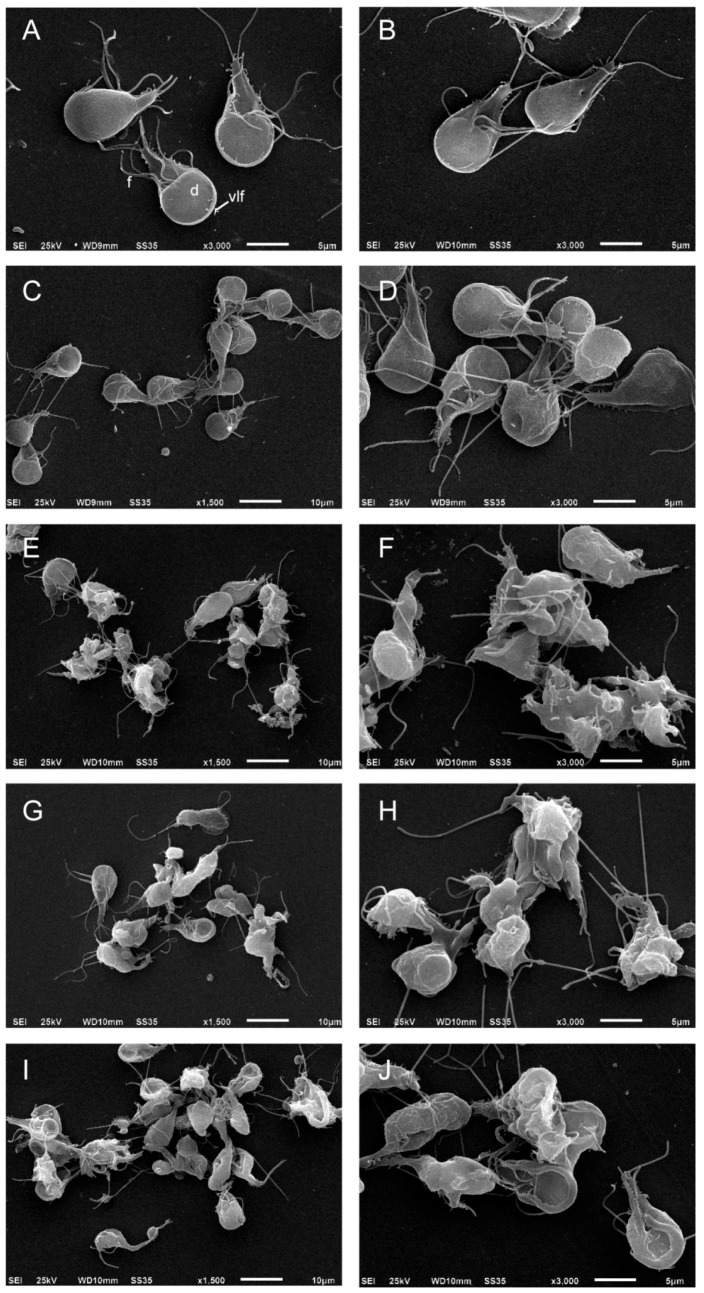
SEM images of trophozoites of *Giardia lamblia* grown in the presence of (**A**) untreated; (**B**) DMSO; (**C**,**D**) burseranin, 24 µM; (**E**,**F**) 5′-desmethoxy-β-peltatin-A-methyl ether, 4 µM; (**G**,**H**) acetyl podophyllotoxin, 2 µM; and (**I**,**J**) podophyllotoxin, 4 µM.

To evaluate the effect of podophyllotoxin-type lignans on the morphology of trophozoites, after 24 h of treatment with DMSO, BUR (24 μM), 5-DES (4 μM), APOD (2 μM), or POD (4 μM), cells were analyzed by scanning electron microscopy (SEM). The images clearly showed morphology changes after drug treatment. Control cells show normal morphology; the ventral disk, flagella, and ventro-lateral flange lack alterations (Figure 5A,B). The BUR treatment did not cause dramatic changes in cell shape; the damage was located principally on the ventro-lateral flange (Figure 5C,D). 5-DES, APOD, and POD produced fairly dramatic changes in morphology; protrusions on the dorsal surface, membrane blebs, disruption of the ventro-lateral flange, damage on the caudal region, and completely misshapen cells (around 70–80%) were observed (Figure 5E–J).

### 2.4. Cytotoxic Effect of Podophyllotoxin-Type Lignans on Human Intestinal Caco-2 Cells

**Figure 6:**
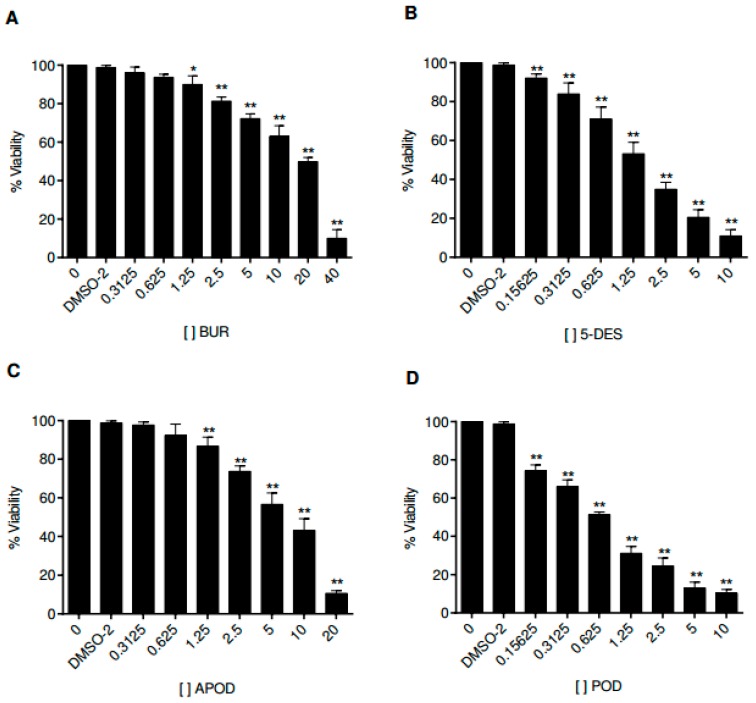
Dose-response curves for cell viability of Caco-2 cells treated with burseranin (**A**), 5′-desmethoxy-β-peltatin-A-methylether (**B**), acetylpodophyllotoxin (**C**), and podophyllotoxin (**D**) by using a typical MTT assay (*p < 0.005, **p < 0.0001).

Cell culture assays have been used to study the life cycle and infection mechanism of *Giardia lamblia* trophozoites and to test the efficacy of therapeutic agents. In this work, we analyzed the effects of the four podophyllotoxin-type lignans on the proliferation of Caco-2 cells. Figure 6 shows the time and dose-dependent alterations in Caco-2 cell growth by all podophyllotoxin-type lignans tested. POD exhibits a more potent activity than 5-DES, APOD, and BUR (IC_50_ 0.65, 2.87, 8.64, and 19.69 μM, respectively) (Table 1).

## 3. Discussion

Drugs commonly used in the treatment of giardiasis produce different results as to their effectiveness, and all of them have undesirable side effects [6,7,8,9]. Additionally, treatment failures have been reported with all of the common anti-*Giardia* agents including metronidazole, quinacrine, furazolidone, and albendazole [27]. Thus, the search for new therapies that are more effective and have fewer side effects is still important. 

Lignans are a group of natural products widely distributed within the plant kingdom, with vast ranges of biological activities. A previous work showed a cytotoxic effect of podophyllotoxin, an aryltetralin-type lignan, against *G. lamblia*; nevertheless, they did not report the possible cell death mechanism involved [17]. In addition, its high cytotoxic effect against mammalian cells renders it unsuitable as an antigiardial agent [28,29]. In searching for new molecules with anti-giardial activity, this study demonstrated the effect of BUR, 5-DES, APOD, and POD, podophyllotoxin-type lignans from *B. fagaroides* var. *fagaroides*, on the viability and morphology of trophozoites of *G. lamblia*. Our results show that 5-DES, APOD, and POD have a dose-dependent effect on trophozoite growth (Figure 1 and Figure 2), cell viability (Figure 3), and adhesion to glass surfaces (Figure 4). For BUR, no significant effects on *Giardia* growth and cell viability were observed. It affects only the adherence, provoking damage on the ventrolateral flange (Figure 4A and Figure 5C,D). The last correlate with Erlandsen et al. [30], they showed that the ventrolateral flange is involved in trophozoite adhesion. Here, it was observed that 5-DES, APOD, and POD produced dramatic changes in cell morphology to different extents; damage on cytoskeleton structures and completely misshapen cells were evident (Figure 5E–L). It is largely known that POD is an anti-tubulin agent; it binds at the interface between α- and β-tubulin, which inhibits the assembly of tubulin into microtubules. In addition, it was recently described that 5-DES and APOD also disrupt microtubule networks in mammalian cells [31]. Considering that microtubules are an essential part of the *Giardia* cytoskeleton, our data suggest that POD, 5-DES, and APOD may inhibit *Giardia* proliferation by perturbing microtubule assembly, and we are currently conducting studies to identify the molecular targets involved. The poor effect observed by BUR in *Giardia* could be explained because BUR is not able to inhibit tubulin assembly, as previously reported [31]. On the other hand, the ability of albendazole to affect *Giardia* trophozoite morphology, adherence, and viability, has been demonstrated in in vitro assays [32]. Our results showed the albendazole toxic effect in *Giardia*, and revealed that APOD was more potent; the cell viability was markedly decreased. 

**Figure 7:**
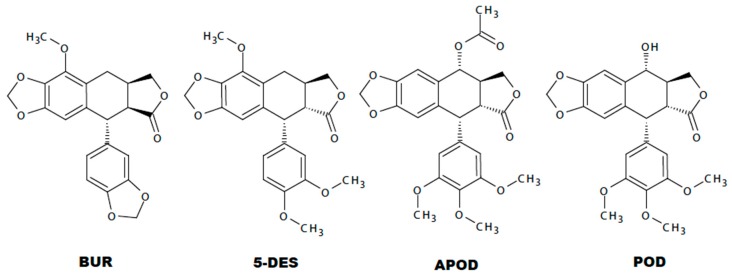
Chemical structure of burseranin (**BUR**), 5′-desmethoxy-β-peltatin-A-methylether (**5DES**), acetylpodophyllotoxin (**APOD**), and podophyllotoxin (**POD**).

Comparing the toxicity of each lignan against *Giardia* trophozoites, the most effective was APOD with an IC_50_ of 2.12 µM followed by POD and 5-DES (IC_50_ 3.88 and 4.53 µM, respectively) (Table 1). The differential lignans’ effects on *Giardia* could establish a structure-activity relationship. The Tanimoto coefficient has been found to be highly effective to demonstrated molecular similarities, and several studies have shown that compounds having structural similarity could present the same activity pattern [33,34,35]. In this study, using a binary strings analysis and Tanimoto coefficient (TC) [36], we compare the structural similarity among BUR, 5DES, APOD, and POD. According to the used descriptors, BUR is the highly-dissimilar lignan to POD (TC 0.22), followed by 5-DES (TC 0.43) and APOD (TC 0.8) (Figure S1 and Table S1). Additionally, several cytotoxicity studies with different podophyllotoxin analogues have established that rings A, B, D, and E from POD are involved in the binding reaction with tubulin (Figure 7) [14,37,38,39]. According to different investigations, the availability of ring A is critical for podophyllotoxin-tubulin binding. Additionally, some studies conclude that a trans-lactone orientation on ring D is related to increased cytotoxic activity along with the methoxyl substituents on the E ring [38,39], the last support the lower activity of BUR.

One of the principal limitations in the search for new drugs to treat giardiasis is the problem of high toxicity on mammalian cells. Therefore, we focused our studies on unraveling the cytotoxic effect of POD, 5-DES, and APOD on the Caco-2 human cell line as a model of the intestinal epithelium. By MTT assay, we identified the concentration- and time-dependent effects of Caco-2 cell’s viability of all lignans tested. The results obtained here demonstrate that the POD was avidly taken up by Caco-2 cells, followed by 5-DES (IC_50_ 0.65 and 2.87 µM, respectively), APOD was clearly less efficiently up-taken by Caco-2 cells (IC_50_ 8.64 µM). The high POD cytotoxicity against mammalian cells has also been reported by other authors (IC_50_ 0.5 µM) [28,29]. Based on selective index (SI), it is desirable to have a high SI, giving maximum antiparasite activity with minimal cell toxicity. The SI data shown in Table 1 indicate that BUR (0.47), 5-DES (0.62), and POD (0.15) are not selective for *Giardia*, they would not be considered for follow up as an antigiardial candidate. In contrast, APOD exhibits a high degree of cytotoxic selectivity (4.1), however, its therapeutic use as an antigiardial candidate is unclear; the SI value is still low compared to drugs of therapeutic use. In conclusion, this is a first investigation showing the potential cytotoxic action of podophyllotoxin-type lignans as antigiardial drugs. Our results support the use of *Bursera fagaroides* as an antidiarrheal treatment in Mexican traditional medicine. Considering the high cytotoxic effect of APOD in trophozoites of *Giardia* and its low toxicity against mammalian cells (SI of 4.1) (Table 1), this compound could possibly represent a promising starting point for structural modifications in the search of new antigiardial drugs.

## 4. Materials and Methods

### 4.1. Podophyllotoxin-Type Lignans from Bursera fagaroides var. fagaroides

The podophyllotoxin-type lignans used in this study, BUR (purity > 96%), 5-DES (purity > 99%), APOD (purity > 99%), and POD (purity > 98%), were provided by Dr. Laura Patricia Alvarez. Briefly, the bark of *B. fagaroides* var. *fagaroides* was collected in the village of Capula between Zacapu and Quiroga, Michoacán, México. Lignan identification was made at the Herbarium of the Instituto Mexicano del Seguro Social (IMSS-12 051) and at the Institute of Botany, Universidad de Guadalajara, México (IBUG-140 748). 

To obtain the lignans, the dry material was processed in the same manner as described previously [22,25]. Briefly, the stem bark of *Bursera fagaroides* var. *fagaroides* was extracted by maceration at room temperature thrice with CH_2_Cl_2_ and fractionated by column chromatography (CC) on silica gel and eluting with n-hexane-EtOAc mixtures, increasing the polarity to yield five fractions: F-1 (1.2 g, 100:00 to 9:1), F-2(2.72 g, 4:1), F-3 (5.1 g, 4:1), F-4 (8.9 g, 7:3), and F-5 (1.6 g, 1:1). F-2 was subjected to CC (90:10 → 70:30, n-hexane/CH_2_Cl_2_) to obtain two fractions. Fractions eluted with n-hexane-CH_2_Cl_2_ (9:1) were chromatographed on silica gel to yield 131 mg of β-sitosterol and 36.5 mg of burseranin (BUR). Fractions eluted with n-hexane-CH_2_Cl_2_ (8:2) were combined and the residue (1.21 g) was purified by column chromatography (90:10 → 00:100, n-hexane/EtOAc) to afford 278.5 mg of acetylpodophyllotoxin (APOD). F-3 was chromatographed on a silica gel column (9:1 → 7:3) with n-hexane/EtOAc. Fractionation resulted in three fractions. The subfraction F-3-3, eluted with 7:3 n-hexane-EtOAc (2.8 g), was purified by silica gel column chromatography, eluting with a gradient of n-hexane/CH2Cl2 (8:2 → 6:4) to yield 117 mg of 5′-desmethoxy-β-peltatin-A-methylether (5-DES). F-4 was subjected to CC and eluted with an isocratic mixture of 65:35 n-hexane/EtOAc, which produced 85 fractions of 100 mL each. Fractions 40–65 were combined and the residue (1.6 g) was adsorbed on reverse phase silica gel and subjected to RP column chromatography and eluted with a gradient of MeOH:H_2_O (1:1 → 6:4) to yield 39 fractions of 50 mL each. Fractions eluted with MeOH-H_2_O (55:45) were purified by silica gel column chromatography, eluting with an isocratic mixture of n-hexane/EtOAc (6:4), to obtain two main fractions. The most polar fraction was submitted to preparative TLC eluted with benzene/EtOAc (55:45) (three developments) to afford 8 mg of podophyllotoxin (POD). All of the isolated compounds were identified using 1D and 2D NMR, optical rotation (OR), and HRMS analyses, and compared with reported values. 

### 4.2. Culture of Giardia lamblia 

Trophozoites of *Giardia lamblia* (WB clone C6) were maintained axenically at 37 °C in borosilicate culture tubes containing Diamond’s TYI-S-33 medium, pH 7.1 [40]. Cultures were maintained by sub-culturing the cells twice a week. 

### 4.3. Cytotoxic Assay and Cell Viability

In order to evaluate the effect of podophyllotoxin-type lignans on *Giardia lamblia* growth, an inoculum of 10,000 cells/mL was exposed to BUR (6, 12, and 24 µM), 5-DES (2 and 4 µM), APOD (1 and 2 µM), or POD (2 and 4 µM) in TYI-S-33 medium for 12, 24, 48, and 72 h at 37 °C. The diluent of the lignans, 0.1% DMSO (Sigma-Aldrich, Saint Louis, MO, USA), and albendazole (IC_50_ 0.5 µM) were used as negative and positive controls, respectively. After the incubation periods, cells were harvested by cooling them in an ice bath and counted using a Neubauer chamber. The cells viability was evaluated using a trypan blue exclusion assay. All experiments were performed by triplicate. Data were analyzed by ANOVA (Graph Pad Prism version 6.01 for Windows, Graph Pad Software, La Jolla, CA, USA) and *p* values of ≤ 0.05 were considered statistically significant.

### 4.4. Adherence Inhibition Assays

To evaluate the effect of podophyllotoxin-type lignans on trophozoites adherence, 10,000 parasites/mL were grown at concentrations and times described above. After incubation, the medium containing non-adherent cells was removed and kept on ice; tubes were filled with cold phosphate-buffered saline (PBS) and placed in an ice bath for 30 min to dislodge the adherent cells. The number of adherent and non-adherent trophozoites was determined by counting in a Neubauer chamber. The results were expressed as percentage of adhered trophozoites in relation to the total number of cells. Experiments were performed in triplicate; the variance was determined using ANOVA (Graph Pad Prism version 6.01 for Windows, Graph Pad Software, La Jolla, CA, USA). 

### 4.5. The Effect of Podophyllotoxin-Type Lignans on Morphology by Scanning Electron Microscopy (SEM) 

To analyze the morphology of trophozoites after the lignans or DMSO treatment, parasites were washed with PBS, fixed with 2.5% glutaraldehyde (Sigma-Aldrich, Saint Louis, MO, USA) in PBS for 1 h, and adhered to 0.1% poly-(ethylenimine) (Sigma-Aldrich, Saint Louis, MO, USA)-coated cover slips. After that, they were fixed in 2% osmium tetroxide (Electron Microscopy Science, Hatfield, PA, USA) for 2 h. Next, cells were washed with PBS, dehydrated in an ascending ethanol serial, subjected to critical-point drying with CO_2_ (Tousimis, Rockville, MD, USA), mounted on stainless steel holders, sputter-coated with a thin layer of gold, and analyzed by SEM (JEOL-JSM6510LV, Tokyo, Japan).

### 4.6. Culture of the Human Intestinal Caco-2 Cells

The human colon carcinoma cell line, Caco-2, was provided by Centro de Investigación Biomédica de Occidente, Guadalajara, México. Cells were cultured at 37 °C in Dulbecco’s modified Eagle’s culture medium (DMEM), supplemented by 10% fetal bovine serum FBS (By Products), in a humidified atmosphere (5% CO_2_ and 95% air). Cells were split twice a week, by detachment with 0.25% Trypsin, 0.025% EDTA (Sigma-Aldrich, Saint Louis, MO, USA), and re-seeding in 25 cm^2^ flasks in a split ratio of 1:4. For experiments, the number of Caco-2 cells per well was estimated by counting cells with an inverted microscope using a Neubauer chamber.

### 4.7. Cell Viability (MTT Assay)

Caco-2 cell viability was evaluated by MTT assay using the tetrazolium dye as a substrate, and conducted according to manufacturer’s protocols (Sigma-Aldrich, Saint Louis, MO, USA). Briefly, Caco-2 cells were seeded in 96 well cell culture plates at a density of 5000 cells/well and pre-incubated at 37 °C for 48 h, before podophyllotoxin-type lignan treatment. The cytotoxicity of each lignan at different serial concentrations was tested. The cells were then treated with DMSO 0.1%, BUR (0.3125, 0.625, 1.25, 2.5, 5, 10, 20, and 40 μM), 5-DES (0.156, 0.3125, 0.625, 1.25, 2.5, 5, and 10 μM), APOD (0.3125, 0.625, 1.25, 2.5, 5, 10, and 20 μM) and POD (0.156, 0.3125, 0.625, 1.25, 2.5, 5, and 10 μM), for 24 h. After the incubation period, the medium was removed and 100 μL of MTT reagent was added to each well, including controls (0.8 mg/mL MTT in serum-free medium), and the cells were incubated at 37 °C for 4 h in an atmosphere of 5% CO_2_. Next, the medium was removed and the formazan crystal formed in living cells was dissolved in 150 µL of DMSO per well. The cell viability was calculated as percent based on the absorbance at 570 nm using a microplate reader (Biochrom, Holliston, MA, USA). Each experiment was performed in triplicate and repeated three different times. The variance was determined using ANOVA (GraphPad Prism version 6.01 for Windows, GraphPad Software, La Jolla, CA, USA). The selectivity index (SI) was calculated as IC_50_Caco-2 cells/IC_50_ parasite.

### 4.8. Structure Similarity Analysis of Podophyllotoxin-Type Lignans

Structure similarities between BUR, 5-DES, APOD, and POD were evaluated using the Tanimoto coefficient (Tc). Briefly, the Tc represents the similarity between two compounds based on the presence or absence of molecular fragments. The Tc will vary from one to zero, a value of zero indicates that no fragments were found to be common to both structures. A value of 1 is reported typically as 100% similarity. In this study, we used ten structural fragments as molecular descriptors (Figure S1), and the Tc of each compound was analyzed and compared to POD (Table S1). For instance, the well-known Tc is given as

$$\mathrm{Tc}=\frac{c}{\left(a+b-c\right)}$$

where *a* represents the bits set in the reference structure, *b* represents the bits set in the enquiry structure, and *c* represents the bits set in common between the reference structure and enquiry structure.

## Figures and Tables

**Table 1:** IC_50_ values of compounds used in this study.

Lignan	*Giardia* IC_50_ µM	Caco-2 IC_50_ µM	Selectivity (IC_50_ Caco-2/IC_50_ *Giardia*)
Burseranin	42.22	19.69	0.47
5′-demethoxy-β-peltatin-A-methylether	4.53	2.87	0.62
Acetylpodophyllotoxin	2.12	8.64	4.1
Podophyllotoxin	3.88	0.65	0.15

## Supplementary Materials

Figure S1: 3-D molecular descriptors utilized in the analysis of similarity. Table S1: Binary strings analysis from each compound according to molecular descriptors and structural similarity coefficients (SSC). The compounds were compared against podophyllotoxin.
