# Supplementary figures and images for: Antigiardial Activity of Podophyllotoxin-Type Lignans from Bursera fagaroides var. fagaroides
Source: Molecules. 2017 May 13;22(5):799. doi: 10.3390/molecules22050799 (PMC6154107; doi:10.3390/molecules22050799)

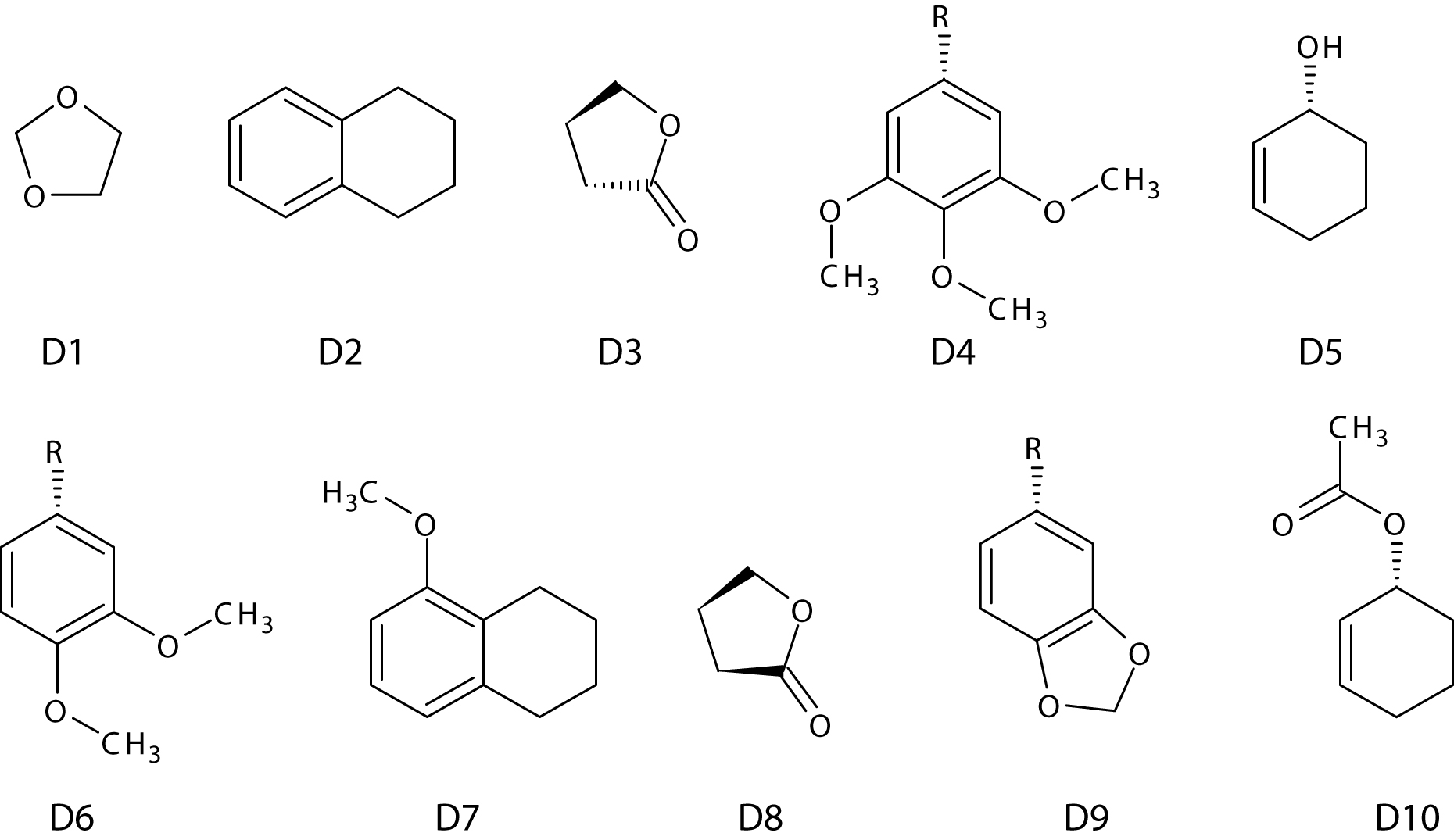

Supplement: Supplementary file 1 [file molecules-22-00799-s001.zip › Figure S1.jpg]

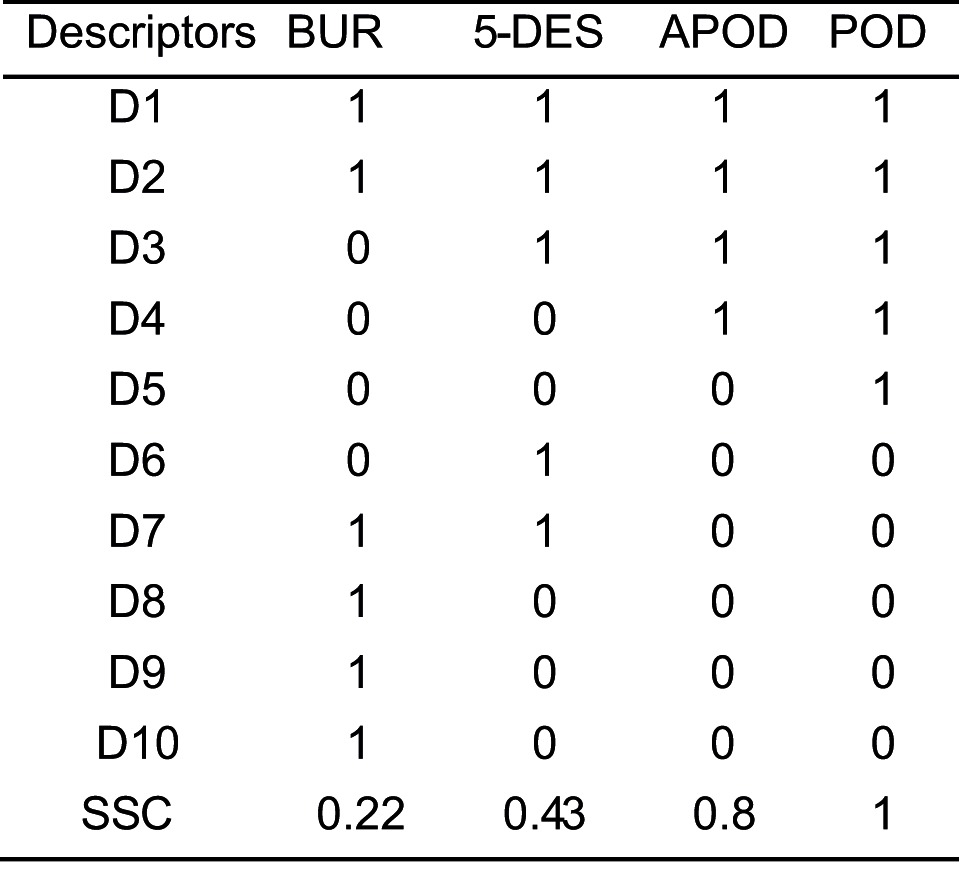

Supplement: Supplementary file 1 [file molecules-22-00799-s001.zip › Table S1.tif]
